# Hemophagocytic Lymphohistiocytosis in the adult critically ill: a narrative review of case reports and case series

**DOI:** 10.3389/fmed.2025.1622770

**Published:** 2025-09-08

**Authors:** Giorgia Montrucchio, Riccardo Traversi, Gabriele Arrigo, Chiara Bonetto, Gabriele Sales, Alessandro Busca, Vito Fanelli, Claudia Filippini, Luca Brazzi

**Affiliations:** ^1^Department of Surgical Sciences, University of Turin, Turin, Italy; ^2^Department of Anesthesia, Intensive Care and Emergency, Anestesia e Rianimazione 1U, Città della Salute e della Scienza Hospital, Turin, Italy; ^3^SSCVD Trapianto Cellule Staminali, Città della Salute e della Scienza Hospital, Turin, Italy

**Keywords:** septic shock, immune dysregulation, hematology critical care, hemophagocytic lymphohistiocytosis (HLH), hemophagocytic syndromes (HPS), HLH-2004 criteria, HScore

## Abstract

**Background:**

Hemophagocytic Lymphohistiocytosis (HLH) is a rare life-threatening syndrome characterized by hyperinflammation caused by abnormally activated macrophages and cytotoxic T cells overlapping with sepsis and multi-organ disfunction (MOD). Its frequency is probably underestimated.

**Methods:**

Patients’ data were extracted from a literature search performed on PubMed (MEDLINE) and EMBASE using the following search terms: “Hemophagocyitic Lymphohistiocytosis” OR “HLH” OR “MACROPHAGE ACTIVATING SYNDROME” OR “MAS” AND “Intensive Care Unit” OR “Critical Care” OR “ICU.” Search was limited to articles published after 2014, when HScore was proposed.

**Results:**

We found 126 case reports and case series for a total of 148 patients with an overall mortality of 47.5%. Main triggers were infections (111 patients; 88.1%) followed by dysimmune disorders (29 patients; 19.7%) and hematological malignancies (20 patients; 13.6%). The following factors were associated with increased ICU mortality: viral infection (76 patients; 52.8%) *p* = 0.0071 and *p* = 0.0086 at multivariate analysis for SARS-CoV-2, hematological malignancies (*p* = 0.0035 at univariate analysis; *p* = 0.0083 at multivariate analysis), invasive mechanical ventilation (116 patients; 83.3%) *p* = 0.0060 at univariate analysis not confirmed in multivariate analysis (*p* = 0.0599). Corticosteroids were associated with reduced ICU mortality at univariate analysis (86 patients; 59.7% *p* = 0.0250) not confirmed at multivariate analysis (*p* = 0.7196).

**Conclusion:**

Evidence from our analysis confirms the severity and rapid evolution of HLH, suggesting the importance of prompt clinical suspicion. Since HLH can be found in different hospital settings, including ICU, we believe that this syndrome should be considered in differential diagnosis for all patients presenting with MOD with unclear etiology. Development of specific diagnostic and therapeutic schemes should be considered a priority.

## Background

Hemophagocytic Lymphohistiocytosis (HLH) is a syndromic disorder characterized by severe hyperinflammation and immune dysfunction with concomitant immune system activation (1).

Although rare, the exact frequency of HLH within the general and critical care population is unknown. However, HLH often requires supportive care management in ICUs (>60% in pediatric HLH cases) with high mortality rates (36–40% of pediatric cases and 41–68% of adult cases) (1).

Classically, HLH is categorized as primary (pHLH) and secondary (sHLH), although significant overlap exists (2). pHLH is caused by genetic mutations inherited in homozygous or compound heterozygous pattern, resulting in disruptive mutations that fully eliminate the function of cytotoxic T cells and NK cells. pHLH is also associated with various immunodeficiency disorders. Anyway, the diagnosis of pHLH cannot be excluded in patients older than 1 year.

Secondary or acquired HLH (sHLH) is induced by triggers such as infection, malignancy, rheumatologic disease, allogenic hematopoietic stem cell transplantation (HSCT), CAR-T therapy (3), drug hypersensitivity or other underlying causes. The immune dysfunction in sHLH is characterized by reversible natural killer (NK) or CD8 + T cell dysfunction, which occurs in some viral infections or rheumatologic disorders, or by NK-cell deficiency, which can occur after chemotherapy or during sepsis.

The diagnosis of HLH in the ICU may be challenging due to lack of specific laboratory, radiologic and histopathologic findings [bone marrow hemophagocytosis may be observed in the absence of proven hemophagocytic syndrome, in particular after blood transfusion or in sepsis (4)], and above all, due to the overlap of its clinical features with the Multi-Organ Disfunction syndrome (MOD) that characterizes the majority of ICU admission.

Scores considering clinical and laboratory finding have been developed for HLH diagnosis and their use is suggested in the ICU setting (4, 5).

Suggested treatment strategies are based on HLH-2004 protocols including high dose corticosteroids, etoposide and cyclosporine, followed by adjunctive/rescue therapy such as intravenous immunoglobulin, plasmapheresis and tocilizumab (1, 5). However, these therapeutic protocols are borrowed from pediatric and hematology guidelines and only scattered studies with data regarding ICU treatment have been reported. A growing interest and novel awareness about this syndrome are spreading among ICU physicians.

Previous observational studies and reviews have been realized in ICU patients in recent years. Table 1 provides an overview of major studies conducted in ICU settings with large cohorts of HLH patients, which offer important perspectives. However, case series and case reports were excluded from the considered literature, although they might have a complementary role in identifying knowledge gaps and generating hypotheses that can inform both clinical practice and future prospective research.

**Table 1:** Major studies on HLH conducted on large cohorts of ICU patients.

Article	Author	Journal	Year	Country	HLH diagnosis and treatment keypoint
High Mortality of HLH in ICU Regardless Etiology or Treatment	Bichon et al.	Frontiers in medicine	2021	France	Mortality of HLH in ICU is high regardless of HLH etiology or treatment
Hemophagocytic Lymphohistiocytosis in Critically Ill Patients	Knaak et al	Shock	2020	Germany	Mortality of HLH in ICU is high, particularly if malignancy-associated
Hemophagocytic lymphohistiocytosis in critically ill patients: diagnostic reliability of HLH-2004 criteria and HScore	Knaak et al.	Critical Care	2020	Gemany	Both HLH-2004 and HScore showed good diagnostic accuracy in critically ill
Hyperferritinemia in Critically Ill Patients	Lachmann et al.	Critical Care Medicine	2020	Germany	Ferritin may improve HLH diagnosis in ICU
Treatment and Mortality of Hemophagocytic Lymphohistiocytosis in Adult Critically Ill Patients: A Systematic Review With Pooled Analysis	Knaak et al.	Critical Care Medicine	2020	Germany	Mortality of HLH in ICU is high. Main trigger is infection

For this reason, here we present results from a literature search on case series and case reports, aimed at describing epidemiology, risk factors, diagnostics test, triggers, treatment modalities and outcomes of HLH patients treated in the ICU. Specifically, our narrative review highlights rare or atypical presentations, diagnostic delays, and treatment challenges that may not be fully captured in large databases or registry-based analyses.

## Methods

### Review search algorithm

Literature search in PubMed (MEDLINE) and EMBASE databases has been performed on June 22, 2024, using the following search terms: (“Hemophagocyitic Lymphohistiocytosis” OR “HLH” OR “Macrophaghe Activation Syndrome” or “MAS”) AND (“Intensive Care Unit” OR “Critical Care” OR “ICU”). Only articles published after 2014—the year of publication of HScore (4), suggested by the Society of Critical Care Medicine for the diagnosis of HLH in intensive care—were taken into consideration.

Given the narrative nature of our work, protocol registration was not undertaken.

### Review study selection

**Figure 1:**
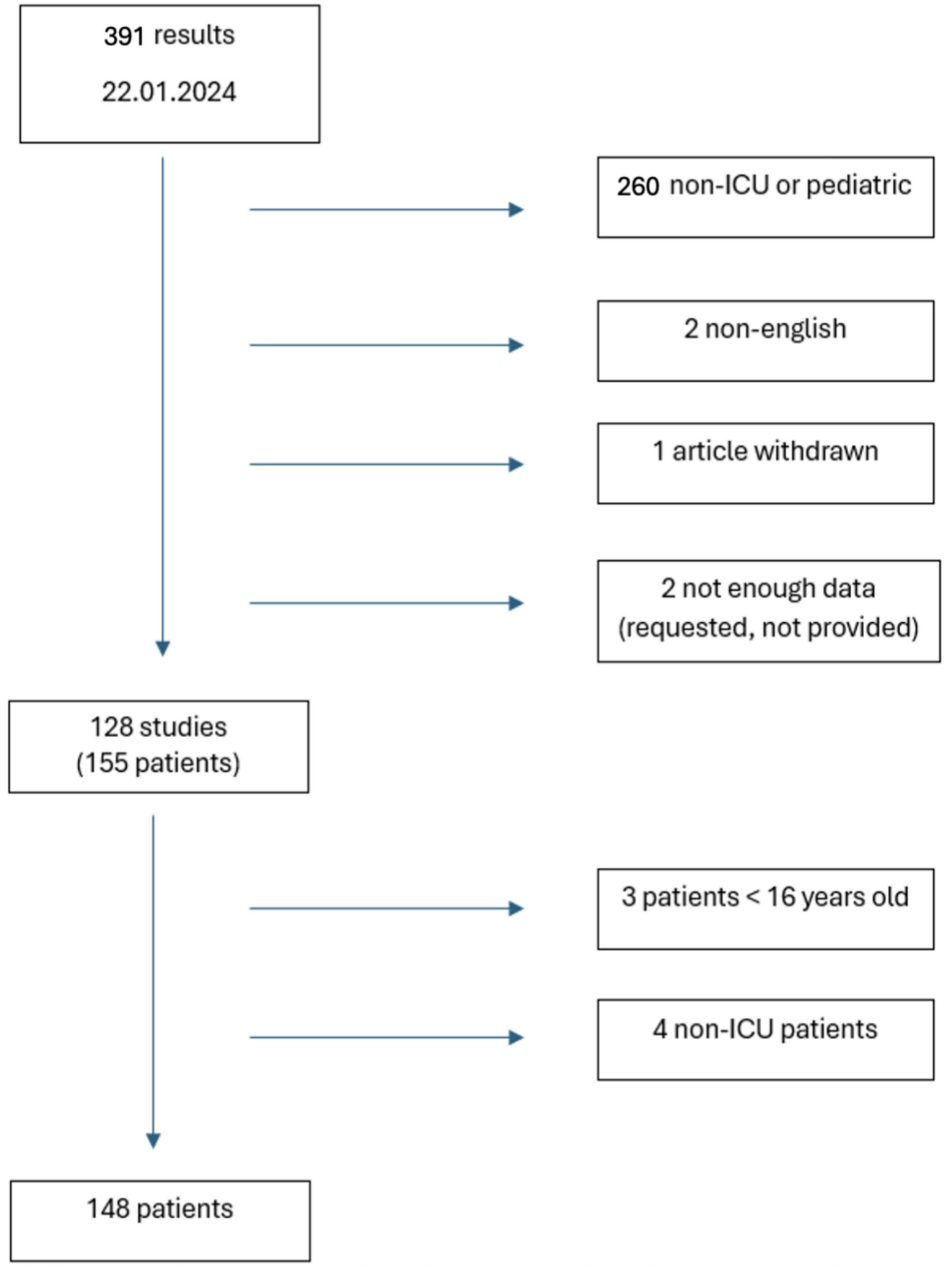
Review search algorithm.

All case reports and case series on patients ≥ 16 years old admitted to ICU for HLH published in English were evaluated (Figure 1). Eligible studies were those including patients with a positive diagnostic score, such as HScore or HLH-2004, for HLH or if HLH diagnosis was reported by paper’s author. In all cases where data regarding clinical course, treatment or outcome were not fully described, corresponding authors were contacted to obtain missing data. Screening of suitable articles was performed manually by two authors who independently reviewed titles and abstracts, with disagreements regarding inclusion in the review was resolved by discussion.

HLH triggers were assigned to predefined categories: viral, bacterial or fungal infection, dysimmune disease, hematologic or solid malignancy, pregnancy related. Data regarding infectious triggers were then divided into sub-categories when the trigger was reported in more than five patients. Specific pharmacological and non-pharmacological treatment was grouped if such treatment was reported in more than five patients. Mortality was categorized into 7-day, 14-day and overall ICU mortality.

### Statistical analysis

Values are presented as means and standard deviation or frequency and proportion. Comparison between groups was made using the t-test or Wilcoxon-Mann-Whitney for and Chi square or Fisher exact test was applied as appropriate. Possible independent risk factors of ICU mortality were tested performing a multivariable logistic model estimating the ODDS RATIO (OR) and the 95% confidence interval (CI 95%). All statistical tests were two-sided and *p* values of 0.05 or less were considered statistically significant. Statistical analyses were conducted using the SAS software package (SAS Institute, Cary, NC; version 9.4).

## Results

### Review and pooled analysis results

Literature search identified 391 papers: 260 were excluded not being conducted in ICU or including pediatric population, 2 not being written in English, 1 being withdrawn and 2 not reporting enough data, even after the investigation with Authors. Fifty-five articles came from North America (43.6%), 35 from Europe (27.8%), 27 from Asia (21.6%), 3 from South America (2.4%), 3 from Middle East (2.4%) and 2 from Africa (1.6%) (Table 2).

**Table 2:** Results from literature search.

Title	Author	Journal	Year of publication	Country	No. of patients
A histopathological observation regarding the possibility of hemophagocytic lymphohistiocytosis in COVID-19 patients (11)	A. Abdollahi	J Gastrointestin Liver Dis	2020	Iran	2
Hemophagocytic lymphohistiocytosis: plasma exchange as a final treatment modality (12)	A. Barosum	Chest	2023	Canada	1
Hemophagocytic lymphohistiocytosis due to acute primary Herpes Simplex Virus 1 infection (13)	A. Drori	J Clin Virol	2015	Israel	1
Helmet mask and tocilizumab for a patient with hemophagocytic lymphohistiocytosis syndrome and COVID-19: a case report (14)	A. Eroglu	Braz J Anesthesiol	2021	Turkey	1
Epstein–Barr-positive classical hodgkin lymphoma-associated haemophagocytic lymphohistocytiosis: a rare case (15)	A. Iardino	BMJ Case Rep	2018	USA	1
Hemophagocytic lymphohistiocytosis and pancreatic cancer: a rare association (16)	A. Jaan	J Community Hosp Intern Med Perspect	2023	USA	1
Febrile conundrum: a case of hemophagocytic lymphohistiocytosis (17)	A. Schenone	Am J Med	2014	USA	1
Perioperative management of recurrent hemophagocytic syndrome in a pregnant woman: a case report (18)	A. Sumii	Am J Case Rep	2023	Japan	1
A rare case of hemophagocytic lymphohistiocytosis mimicking flare of systemic lupus erythematosus (19)	A. Tiwana	Respirol Case Rep	2023	Pakistan	1
Acute Cytomegalovirus (CMV) infection associated with hemophagocytic lymphohistiocytosis (HLH) in an immunocompetent host meeting all eight HLH 2004 diagnostic criteria (20)	AK Bonnecaze	Cureus	2017	USA	1
Haemophagocytic syndrome and paradoxical reaction to tuberculostatics after treatment with infliximab (21)	AT Mariño	Farm World Sci	2010	Spain	1
From upper respiratory symptoms to hemophagocytic lymphohistiocytosis: case report of a human adenovirus infection in haploidentical hematopoietic stem cell transplant recipient (22)	B. Demey	Patogens	2021	France	1
Acute abdomen and severe lactic acidosis can lead to a surprising diagnosis (23)	B. Jung	Intensive Care Med	2010	France	1
Natural killer/T-cell lymphoma and secondary haemophagocytic lymphohistiocytosis in pregnancy (24)	B. Neistadt	BMJ Case Rep	2018	USA	1
Hemophagocytic lymphohistiocytosis in a patient with COVID-19 treated with Tocilizumab: a case report (25)	B. Tholin	J Med Case Rep	2020	Norway	1
Secondary hemophagocytic lymphohistiocytosis: a challenging diagnosis in a patient with autoimmune hepatitis (26)	C. Casault	Case Rep Crit Care	2019	Canada	1
Rescue of cytokine storm due to hlh by hemoadsorption in a CTLA4-deficient patient (27)	C. Greil	J Clin Immunol	2017	Germany	1
A fatal case of delayed diagnosis with hemophagocytic lymphohistiocytosis (28)	C. Li	Chest	2023	China	1
When COVID-19 and HIV join forces to trigger hemophagocytic lymphohistiocytosis (29)	C. Ramirez	Chest	2023	Chile	1
Catastrophic hemophagocytic lymphohistiocytosis in a young man with nephrotic syndrome (30)	CC Chang	Clin Chim Acta	2023	China	1
Continuous renal replacement therapy with Oxiris^®^ membrane in severe ebstein-barr virus-mediated hemophagocytic lymphohistiocytosis: a case report (31)	CN Samman	Blood Purif	2021	Canada	1
Flea-borne typhus causing hemophagocytic lymphohistiocytosis: an autopsy case (32)	D. Chandramohan	Infect Dis Rep	2023	USA	1
Hemophagocytic lymphohistiocytosis associated to *Haemophilus parainfluenzae* endocarditis– a case report (33)	D. I. Costescu Strachinaru	Acta Clin Belg	2017	Belgium	1
Fatal mucormycosis and aspergillosis coinfection associated with haemophagocytic lymphohistiocytosis: a case report and literature review (34)	D. Loubet	J Mycol Med	2023	France	1
Haemophagocytic lymphohistiocytosis in an adult with postacute COVID-19 syndrome	D. Wiseman	BMJ Case Rep	2021	Canada	1
Case report: disseminated Herpes simplex virus 1 infection and hemophagocytic lymphohistiocytosis after immunomodulatory therapy in a patient with coronavirus disease 2019 (35)	E. Mazzotta	Front Med (Lausanne)	2022	USA	1
A 58-year-old man with acute encephalopathy, fever, and multi-organ dysfunction (36)	E. Rogers	Chest	2020	USA	1
Fatal hemophagocytic lymphohistiocytosis in a patient with *Miliary tuberculosis*: a case report (37)	EE Schnippers	SN Compr Clin Med	2022	Netherlands	1
Hemophagocytic lymphohistiocytosis complicated by multiorgan failure: a case report (38)	F. Lovisari	Medicine (Baltimore)	2017	Italy	1
A rare case of acute respiratory distress syndrome in a young adult with hemophagocytic lymphohistiocytosis and systemic EBV-positive T-cell lymphoma of childhood (39)	F.N.U. Chesta	Chest	2023	USA	1
Fatal septic shock in a patient with hemophagocytic lymphohistiocytosis associated with an infectious mononucleosis (40)	G. Berlot	Case Rep Crit Care	2018	Italy	1
Macrophage activation in COVID-19 patients in intensive care unit (41)	G. Labro	J Med Cases	2020	France	6
Hemophagocytic lymphohistiocytosis in unspecific virus infection (42)	G. Lachmann	Anaesthesist	2019	Berlin	1
Hemophagocytic lymphohistiocytosis in renal transplant recipients: a 2-case report (43)	G. Mascia	Transplant Proc	2020	Italy	1
Hemophagocytic lymphohistiocytosis of comorbid etiology: a clinical dilemma (44)	G. Nader	Chest	2023	Sint Marteen	1
How to survive the (cytokine) storm (45)	H. Abu-Hashish	Chest	2023	Israel	1
Hemophagocytic lymphohistiocytosis and relapsing polychondritis with acute myelogenous leukemia: case report and review of the literature (46)	H. Alsaid	Am J Case Rep	2020	Norway	1
A case of hemophagocytic lymphohistiocytosis induced by acute human immunodeficiency virus infection (47)	H. Sheikh	Chest	2023	USA	1
A man in his forties with increasing shortness of breath (48)	H. Tveiten	Tidsskr Nor Laegeforen	2020	Greece	1
An unusual triad of hemophagocytic syndrome, lymphoma and tuberculosis in a non-HIV patient (49)	HRT Hashimi	Am J Case Rep	2017	Netherlands	1
Fulminant Epstein–Barr virus-associated hemophagocytic syndrome in a renal transplant patient and review of the literature (50)	I Romiopoulos	Transpl Infect Dis	2016	USA	1
Case report: a fatal combination of hemophagocytic lymphohistiocytosis with extensive pulmonary microvascular damage in COVID-19 pneumonia (51)	J H von der Thüsen	J Hematop	2021	S. Korea	1
A case of *Miliary tuberculosis* causing hemophagocytic lymphohistiocytosis leading to multiorgan dysfunction (52)	J. Giurnitano	Chest	2023	USA	1
Postpartum hemophagocytic lymphohistiocytosis: a case report (53)	J. Ho Han	World J Clin Cases	2023	USA	1
Rare case of refractory hypoxia and severe multiorgan failure from secondary lymphohistiocytosis successfully bridged to treatment with extracorporeal membrane oxygenation support (54)	J. Hundal	Indian J Crit Care Med	2022	USA	1
Parvovirus B19-induced hemophagocytic lymphohistiocytosis: case report and review of the literature (55)	J. Kalmuk	Clin Case Rep	2019	USA	1
Haemophagocytic lymphohistiocytosis in pregnancy: a pertinent case during the COVID-19 pandemic (56)	J. Pott	BMJ Case Rep	2021	USA	1
Acute respiratory distress syndrome secondary to histoplasmosis-induced hemophagocytic lymphohistiocytosis (57)	JA Huapaya	Arch Bronconeumol (Engl Ed)	2019	Spain	1
Simultaneous acquired self-limited hemophagocytic lymphohistiocytosis and Kikuchi necrotizing lymphadenitis in a 16-year-old teenage girl: a case report and review of the literature (58)	JA Sykes	Pediatr Emerg Care	2016	USA	1
Case report: cytokine hemoadsorption in a case of hemophagocytic lymphohistiocytosis secondary to extranodal NK/T-cell lymphoma (59)	JC Ruiz-Rodríguez	Front Med (Lausanne)	2022	UK	4
Case report: secondary hemophagocytic lymphohistiocytosis with disseminated infection in chronic granulomatous disease-a serious cause of mortality (60)	JD Squire	Front Immunol	2020	USA	1
Haemophagocytic lymphohistiocytosis in a patient with severe burns (61)	JRS Porter	Anesthesia	2013	USA	1
Lamotrigine-induced hemophagocytic lymphohistiocytosis with takotsubo cardiomyopathy: a case report (62)	JY Zhou	J Med Case Rep	2019	Belgium	1
A 32-year-old man with hiv infection, pleural effusions, and lymphadenopathy (63)	JZ Xian	Chest	2018	USA	1
EBV-associated hemophagocytic lymphohistiocytosis complicated by severe coagulation disorders and opportunistic infections: case report of a survivor (64)	K. Saevels	Clin Case Rep	2018	China	1
A rare case of hemophagocytic lymphohistiocytosis-induced by adult-onset Still’s disease (65)	K. A. Roth	Chest	2023	Belgium	2
Potential killer in the ICU—severe tuberculosis combined with hemophagocytic syndrome	L. Chen	Medicine (Baltimore)	2017	USA	1
Biallelic mutations in the cfhr genes underlying atypical hemolytic uremic syndrome in a patient with catastrophic adult-onset still’s disease and recurrent macrophage activation syndrome: a case report (66)	L. Dillemans	Clin Immunol	2023	Switzerland	1
Hemophagocytic lymphohistiocytosis complicated by acute respiratory distress syndrome and multiorgan failure (67)	L. Liu	J Investig Med High Impact Case Rep	2021	China	1
EBV-positive large B-cell lymphoma with an unusual intravascular presentation and associated haemophagocytic syndrome in an hiv-positive patient: report of a case expanding the spectrum of ebv-positive immunodeficiency-associated lymphoproliferative disorders (68)	L. Veloza	Virochows Arch	2022	USA	1
Case report: hemophagocytic lymphocytosis in a patient with glutaric aciduria type IIC (69)	L. Wang	Front Immunol	2022	USA	1
Typhoid fever complicated by hemophagocytic lymphohistiocytosis and rhabdomyolysis (70)	LR Non	Am J Trop Med Hyg	2015	USA	1
A case of recurrent pregnancy-induced adult onset familial hemophagocytic lymphohistiocytosis (71)	LY Wang	World J Oncol	2018	USA	1
Secondary hemophagocytic lymphohistiocytosis in the setting of metastatic renal cell carcinoma: a case report (72)	M El-Masry	J Med Case Rep	2017	USA	1
Curious case of secondary HLH triggered by HIV infection mimicking sepsis (73)	M Gao	Chest	2023	USA	1
Macrophage activating syndrome causing decompensated right heart failure (74)	M. Chizinga	Respir Med Case Rep	2021	Tunisie	1
Anaplasmosis-induced hemophagocytic lymphohistiocytosis (75)	M. de Jesus	Proceedings (Baylor University. Medical Center)	2022	Brazil	1
Haemophagocytic lymphohistiocytosis induced by A/H1N1 influenza (76)	M. Dlela	Presse Med	2019	Ireland	1
Hemophagocytic syndrome in a patient with hiv and histoplasmosis: a not so rare correlation (77)	M. Freire	Clin Patol	2022	Spain	2
Haemophagocytic lymphohistiocytosis (HLH)-associated stress cardiomyopathy secondary to autoimmune conditions successfully treated with anakinra (78)	M. Khalid	BMJ Case Rep	2021	Belgium	1
Disseminated tuberculosis and hemophagocytic syndrome although tb prophylaxis in patients with inflammatory bowel disease treated with infliximab (79)	M. Martinez-Pillado	ID Cases	2019	Tunisie	1
Case report: hemorrhagic fever with renal syndrome presenting as hemophagocytic lymphohistiocytosis (80)	M.A.J. De Smet	Front Med (Lausanne)	2022	USA	1
Hemophagocytic syndrome in a pregnant renal transplant recipient associated with Cytomegalovirus infection (81)	MB Salem	Exp Clin Transplant	2021	India	1
A 19-year-old college student with headache, photophobia, and flulike illness (82)	MR Mourad	Chest	2017	USA	1
Sepsis of unknown origin with multiorgan failure syndrome: think of hemophagocytic lymphohistiocytosis (83)	N Maheshwari	Indian J Crit Care Med.	2015	India	1
Diagnosis of GATA2 deficiency in a young woman with hemophagocytic lymphohistiocytosis triggered by acute systemic cytomegalovirus infection (84)	N. Burak	Am J Case Rep	2021	Malasya	1
Epstein–Barr virus infection-related hemophagocytic lymphohistiocytosis (85)	N. Kumar	Indian J Crit Care Med	2015	Switzerland	1
Pregnancy-related hemophagocytic lymphohistiocytosis associated with cytomegalovirus infection: a diagnostic and therapeutic challenge (86)	N. Tumian	Taiwan J Obstet Gynecol	2015	USA	1
Haemophagocytic lymphohistiocytosis and liver failure-induced massive hyperferritinaemia in a male COVID-19 patient (87)	NM Zelwegger	Swiss Med Wkly	2021	Greece	1
High-grade fever and pancytopenia in an adult patient with common variable immune deficiency (88)	P. Bajaj	Allergy Asthma Proc	2014	USA	1
Hemophagocytic lymphohistiocytosis syndrome associated with Epstein–Barr infection in an immunocompetent patient. A case study (89)	P. Ioannou	Germs	2020	Germany	1
Hemophagocytic lymphohistiocytosis: a rare complication of COVID-19 in a patient with sickle cell anemia (90)	R. Al-Handola	Cureus	2023	USA	1
Adult hemophagocytic lymphohistiocytosis causing multi organ dysfunction in a patient with multiple autoimmune disorders: when the immune system runs amok (91)	R. Fleishmann	Clin Case Rep	2015	USA	1
An overlap of septic shock and hemophagocytic lymphohistiocytosis: a diagnostic challenge in the intensive care unit (92)	R. Jariwal	Chest	2023	USA	1
Diffuse Large B-cell Lymphoma with secondary hemophagocytic lymphohistiocytosis presenting as acute liver failure (93)	R. Patel	ACG Case Rep J	2017	Spain	3
Hemophagocytic lymphohistiocytosis: a potentially underrecognized association with systemic inflammatory response syndrome, severe sepsis, and septic shock in adults (94)	R. Raschke	Chest	2011	USA	2
Hemophagocytic lymphohistiocytosis/macrophage activation syndrome (HLH/MAS) following treatment with tisagenlecleucel (95)	RM Martin-Rojas	Clin Case Rep	2022	Switzerland	1
Macrophage activation syndrome in a patient with Systemic Lupus Erythematosus (SLE) and dual viremia (96)	S. Awasthi	J Community Hosp Intern Med Perspect	2020	Germany	1
Hemoadsorption treatment with cytosorb^®^ in probable hemophagocytic lymphohistiocytosis: a role as adjunctive therapy? (97)	S. Ceruti	Case Rep Hematol	2021	Germany	2
Cytokine adsorption is a promising tool in the therapy of hemophagocytic lymphohistiocytosis (98)	S. Frimmel	Int J Artif Organs	2019	Japan	1
First description of single-pass albumin dialysis combined with cytokine adsorption in fulminant liver failure and hemophagocytic syndrome resulting from generalized herpes simplex virus 1 infection (99)	S. Frimmel	Liver Transpl	2014	USA	1
A case of hemophagocytic lymphohistiocytosis in a hemodialysis patient with Coronavirus disease 2019 (100)	S. Kaneko	CEN Case Rep	2023	USA	1
Severe COVID-19-induced hemophagocytic lymphohistiocytosis (101)	S. Khan	Cureus	2023	India	1
An intriguing presentation of Epstein–Barr virus-associated hemophagocytic lymphohistiocytosis (102)	S. Quadri	Cureus	2020	Austria	1
Visceral Leishmaniasis associated hemophagocytic lymphohistiocytosis – case report and systematic review (103)	S. Rajagopala	J Infect	2008	Japan	3
Hemophagocytic lymphohistiocytosis in COVID-19 case reports of a stepwise approach (104)	S. Schnaubelt	Medicine (Baltimore)	2021	France	1
Hemophagocytic syndrome with severe sepsis caused by *capnocytophaga canimorsus* (105)	S. Terashima	Am J Emerg Med	2020	Taiwan	1
HHV8-related hemophagocytic syndrome: diagnosis is in the eye (106)	S. Valade	Intensive Care Med	2018	USA	1
*Miliary tuberculosis*-related acute respiratory distress syndrome complicated with hemophagocytic lymphohistiocytosis syndrome (107)	SJ Shiu	Case Rep Infec Dis	2019	USA	1
Cytomegalovirus pneumonitis-induced secondary hemophagocytic lymphohistiocytosis and SIADH in an immunocompetent elderly male literature review (108)	SM Patil	ID Cases	2020	Japan	1
An unusual case of macrophage activation syndrome (MAS)—hemophagocytic lymphohistiocytosis (HLH) triggered by necrotizing autoimmune myopathy (109)	T. Brown	Cureus	2023	USA	1
Fatal case of TAFRO syndrome associated with over-immunosuppression: a case report and review of the literature (110)	T. Matsuhisa	Nagoya J Med Sci	2019	USA	3
What intensivists need to know about hemophagocytic syndrome: an underrecognized cause of death in adult intensive care units (111)	T. Okabe	J Intensive Care Med	2011	China	1
Epstein–Barr virus–associated hemophagocytic syndrome mimicking severe sepsis (112)	T. Spivack	J Emerg Trauma Shock	2008	USA	1
Hemophagocytic lymphohistiocytosis is associated with *Bartonella Henselae* infection in a patient with multiple susceptibility genes (113)	T. Yang	Ann Clin Microbiol Antimicrob.	2020	USA	1
Hemophagocytic lymphohistiocytosis and Hodgkin Lymphoma in a newly diagnosed hiv patient: a diagnostic dilemma (114)	TJ Okobi	Cureus	2023	France	1
Ehrlichiosis presenting as hemophagocytic lymphohistiocytosis in an immunocompetent adult (115)	TP Patel	ID Cases	2020	USA	1
Life-threatening hemophagocytic syndrome triggered by disseminated toxoplasmosis in a young patient with previously unknown AIDS (116)	V. Guiraud	Rev Med Interne	2022	USA	3
A case series of endemic infections associated hemophagocytic lymphohistiocytosis (HLH) mimicking severe sepsis syndrome	V. Kollipara	Respir Med Case Rep	2019	France	1
Case of haemophagocytic lymphohistiocytosis following Epstein–Barr virus infection (117)	V. Krakowski	BMJ Case Rep	2021	USA	1
Management of hemophagocytic lympho-histiocytosis in critically ill patients (118)	V. Lemiale	J Intensive Care Med	2020	S. Korea	1
Reading between the lines: hemophagocytic lymphohistiocytosis with concurrent neuroleptic malignant syndrome triggered by a drug–drug interaction (119)	V. Turbay Caballero	Chest	2023	China	1
Secondary hemophagocytic lymphohistiocytosis associated with heat stroke: a case report and review of literature (120)	W. Wi	Medicine (Baltimore)	2023	China	1
Acute fibrinous and organizing pneumonia complicated with hemophagocytic lymphohistiocytosis caused by chronic active epstein–barr virus infection: a case report (121)	X. Wu	BMC Infect Dis	2021	USA	1
Malaria-associated secondary hemophagocytic lymphohistiocytosis: a case report (122)	X. Zhou	World J Clin Cases	2021	China	2
Hemophagocytic lymphohistiocytosis secondary to disseminated histoplasmosis in rheumatologic disease (123)	Y Kusne	Case Rep Crit Care	2021	Japan	1
Case report: a case of sepsis caused by Rickettsial infection-induced hemophagocytic syndrome (124)	Y. Cao	Front Med (Lausanne)	2023	Japan	1
A case of complete atrioventricular block in secondary hemophagocytic syndrome/hemophagocytic lymphohistiocytosis recovered by plasma exchange and cytokine absorbing therapy with AN69ST continuous hemodiafiltration (125)	Y. Harano	Immunol Med	2020	USA	1
A case of hemophagocytic lymphohistiocytosis after the primary epstein–barr virus infection (126)	Y. Kitazawa	Clin Appl Thromb Hemost	2007	China	1
Conservative treatment failure in Epstein–Barr virus-induced hemophagocytic lymphohistiocytosis (127)	Y. Park	Chest	2023	China	1
Novel swine-origin influenza a (H1N1) virus-associated hemophagocytic syndrome--a first case report (128)	Y. Zheng	Am J Trop Med Hyg	2010	Taiwan	1
Disseminated tuberculosis associated hemophagocytic lymphohistiocytosis in a pregnant woman with Evans syndrome: a case report and literature review (129)	YF Shi	Front Immunology	2021	China	1
Extensive community-acquired pneumonia with hemophagocytic syndrome caused by *Aeromonas Veronii* in an immunocompetent patient (130)	YH Ku	J Microbiol Immunol Infect	2017	USA	1
Successful treatment of mycobacterial infection associated hemophagocytic lymphohistiocytosis with etoposide and anti-tuberculous therapy: a case report (131)	YH Wang	BMC Infect Dis	2020	China	1
Hemophagocytic syndrome masquerading as septic shock: an approach to such dilemma (132)	Z. Hindi	SAGE Open Med Case Rep.	2017	USA	1
Hemophagocytic lymphohistiocytosis associated to *Klebsiella pneumoniae* infection: a case report (133)	Z. Zhang	Front Immunol	2021	China	3
Hemophagocytic lymphohistiocytosis in the elderly (134)	R. Altook	Am J Med Sci	2019	USA	1
A case of secondary hemophagocytic lymphohistiocytosis in a patient with SLE following COVID-19 infection and babesiois (135)	M. Ali	Chest	2023	USA	1

Overall, data on suspected triggers, diagnostic workup, treatment and mortality were available for 146 patients with a mean age of 45 [±17] years.

Overall mortality was 47.5%: 25 (19.2%) and 30 (23.8%) patients died before 7 and 14 days from ICU admission, respectively. Age was significantly higher in non-survivors (*p* = 0.0251) at univariate analysis (Table 3) but not at multivariate analysis (Table 4).

**Table 3:** Results from univariate analysis of ICU mortality.

Variable	Total (*N* = 148)	A (*N* = 74)	D (*N* = 67)	*p*-value
Age, yr, mean (std. dev.)	45 (17)	41.4 (16)	48 (8)	**0.0251**
Sex (M) *n* (%)	80 (55.6)	35 (48.0)	45 (63.4)	0.0624
Bacterial infection *n* (%)	25 (17.0)	14 (18.9)	11 (15.1)	0.5344
Viral infection *n* (%)	76 (52.8)	31 (41.9)	45 (64.31)	**0.0071**
Fungal infection *n* (%)	6 (4.0)	4 (5.4)	2 (2.7)	0.6810
EBV *n* (%)	29 (19.7)	13 (17.6)	15 (21.9)	0.5075
CMV *n* (%)	8 (5.4)	7 (17.6)	1 (1.4)	0.0630
SARS CoV 2 *n* (%)	21 (14.3)	5 (6.8)	16 (21.9)	**0.0086**
HIV *n* (%)	8 (5.4)	3 (4.1)	5 (6.9)	0.4940
HSV1 *n* (%)	8 (5,4)	2 (2.7)	6 (8.2)	0.1662
*Histoplasma capsulatum n* (%)	6 (4.0)	4 (5.4)	2 (2.7)	0.6810
*M. tuberculosis n* (%)	8 (5.4)	7 (9.6)	1 (1.4)	0.0629
Pregnancy related *n* (%)	7 (4.8)	5 (6.9)	2 (2.7)	0.4416
Disimmune disease *n* (%)	29 (19,7)	18 (24.3)	11 (15.1)	0.1585
Hematologic malignancy *n* (%)	20 (13.6)	4 (5.4)	16 (21.9)	**0.0035**
Solid neoplasm *n* (%)	4 (2.7)	1 (1.4)	3 (4.1)	0.3664
Biopsy *n* (%)	106 (73.1)	62 (83.8)	44 (62.0)	**0.0031**
IMV *n* (%)	116 (82.3)	53 (73.6)	63 (91.3)	**0.0060**
ECMO *n* (%)	7 (5.2)	6 (8.2)	1 (1.6)	0.1240
CRRT *n* (%)	52 (38.2)	23 (46.0)	27 (54.0)	0.1053
High dose steroid *n* (%)	86 (59.7)	49 (69.0)	37 (50.7)	**0.0250**
Etoposide *n* (%)	35 (24.0)	20 (27.4)	15 (20.6)	0.3324
Tocilizumab *n* (%)	6 (4.1)	3 (4.1)	3 (4.1)	0.9999
Anakinra *n* (%)	11 (16.4)	7 (10.0)	4 (5.6)	0.3591
IVIG *n* (%)	24 (16.2)	14 (18.9)	10 (13.9)	0.4123
Penthaglobin^®^ *n* (%)	3 (2.0)	1 (1.4)	2 (1.7)	0.6198
Plasmapheresis *n* (%)	5 (3.4)	2 (2.7)	3 (4.1)	0.6810
Cyclosporine *n* (%)	9 (6.2)	4 (5.4)	5 (6.9)	0.7452

A: alive; D: dead; EBV: Epstein–Barr Virus; CMV: Citomegalovirus; HIV: Human Immunodeficency Virus; HSV1: Herpes Simplex Virus type 1; IMV: Invasive Mechanical Ventilation; ECMO: Extracorporeal Membrane Oxygenator; CRRT: Continuous Renal Replacement Therapy. Bold numbers shows statistically significant *p*-values.

**Table 4:** Results of multivariable analysis of ICU mortality.

Variable	OR (95% CI)	*p*-value
Age, years	1.008 (0.984–1.033)	0.5161
Sex (M)	0.785 (0.348–1.770)	0.5589
Viral infection	**2.341 (1.050–5.223)**	**0.0377**
Hematologic malignancy	**6.916 (1.645–29.079)**	**0.0083**
Biopsy	**0.227 (0.087–0.591)**	**0.0024**
Invasive mechanical ventilation	3.198 (0.953–10.735)	0.0599
High dose steroid	0.853 (0.359–2.027)	0.7196

Bold numbers shows statistically significant *p*-values.

Diagnosis was performed using the composite score (HLH-2004, HScore) in 78 (52.70%) patients; clinical suspicion or direct hematology referral in 62 (41.9%) cases and was only autoptic in 6 (4.05%) cases.

Bone marrow biopsy (BMB) was performed on 108 (74.0%) patients and this subgroup was found to have a lower ICU mortality (*p* = 0.003).

### Triggers

The most frequent suspected triggers were infections (106), followed by dysimmune disorders (30), malignancies (24), HIV (8) and pregnancy (7). Seventy-six patients had viral infection, 24 had bacterial infection and 6 fungal infections.

The most represented pathogens were Epstein–Barr virus (EBV, 29; 19.7%), SARS-CoV-2 (21; 14.3%) and Citomegalovirus (CMV, 8; 5.4%).

Viral infection was found to have a significant correlation with ICU mortality (*p* = 0.0377) at multivariate analysis.

The trigger factor was found to be hematologic malignancy in 20 (13.6%) patients and malignancy overall in 24 (16.3%).

All patients with human immunodeficiency virus (HIV) (8; 5.4%) had clinical and/or laboratory criteria defining acquired immunodeficiency syndrome (AIDS).

ICU mortality was found to be significantly correlated with viral infection (*p* = 0.0071) and SARS-CoV-2 (*p* = 0.0086) at univariate analysis. Hematologic malignancy was also statistically correlated with mortality at both univariate (*p* = 0.0035) and multivariate (*p* = 0.0083) analysis.

### Treatment

One hundred and sixteen patients (82.3%) were treated with invasive mechanical ventilation, 52 required continuous renal replacement therapy (CRRT) (38.2%), 7 (5.2%) extracorporeal membrane oxygenator (ECMO) namely veno-venous ECMO in 6 patients and veno-arterial ECMO for cardiogenic shock in 1 patient.

Invasive mechanical ventilation was statistically associated with ICU mortality at univariate analysis (*p* = 0.0060) but not at multivariate analysis (*p* = 0.0599).

The most used pharmacological treatment were corticosteroids (86 cases; 59.7%), etoposide (35; 23.6%), intravenous immunoglobulin (IVIG) (24; 16.2%). Only corticosteroids use was associated with a reduction in ICU mortality (*p* = 0.0250) at univariate analysis but not at multivariate analysis (*p* = 0.7196).

## Discussion

HLH is an under-recognized, life-threatening syndrome requiring prompt but challenging diagnosis and treatment, especially in the ICU context. Although limited data exist on critically ill adult with HLH, in recent years there has been a growing interest in understanding the epidemiology and outcomes of this condition (1, 6). A large retrospective study conducted in a French ICU and a German pooled analysis already showed clinical relevance of HLH in intensive care setting, highlighting most relevant triggers and clinical features of critically ill patients with HLH.

In this paper we reviewed the evidence from 126 case reports and case series that analyzed cases of HLH in the ICU.

Most of the articles included in our analysis come from North America and Europe and this geographical distribution could be explained by the poor availability of clinical and laboratory resources in low-and middle-income countries.

Included patients were mainly male, in line with data reported in the literature (1, 7).

The mortality we observed in this analysis is also in line with the values reported in other studies on severe patients with HLH without any significant differences being observed between the genders.

Although age at the univariate analysis seems to be correlated with mortality, the limited sample size probably did not allow us to confirm the data at the multivariate analysis. On the other hand, viral infections appear to explain a significant increase in mortality and among viral infections those induced by SARS-CoV-2 seem to be particularly important, probably due to the intense inflammatory pattern brought about by COVID-19. It should also be considered that the growing interest in HLH in recent years coincides with the pandemic period and the growing research activity related.

Viral infection, and particularly that induced by EBV, appears to have a more significant impact than reported by other works conducted both in ICU and outside (6, 7). However, it was not possible to identify a statistically higher mortality in the EBV group.

We observed a frequency of autoimmune disease in line with data reported in the literature. Hematologic malignancy accounted for a small share of triggers (12,2%) even in comparison with other studies on ICU and non-ICU population (6, 7), but strongly correlated with higher mortality. Hematologic patients requiring ICU admission are burdened with a constant increase in mortality regardless of diagnosis (8). In this context HLH in patients with hematologic malignancy can be seen as the progression of patients characterized by massive immunological dysregulation to multi-organ failure.

Almost two-third of patients underwent BMB or biopsy of other immune tissues and this cohort showed a significantly lower mortality. It can be assumed that this confirms the role of histopathology as a fundamental criterion used by clinicians to establish diagnosis and start a specific treatment for HLH. However, it should be noted that also composite scores (especially HScore) proved to be able to correctly guide clinical decision with sufficiently good sensitivity and specificity.

No treatment, except for high dose corticosteroids, has been shown to significantly reduce mortality and even this efficacy has not been confirmed by multivariate analysis or found in other studies.

This could be explained by at least two reasons: first, HLH treatments – both pharmacological and non-pharmacological – lack of standardization and the choice of their use is left to single centers expertise, second, all these therapies draw their efficacy from hematology setting and may not be as effective for critically ill patients. Aside from standard treatments suggested by available guidelines based on corticosteroids and etoposide as first-line therapy, Ruxolitinib, a Janus Kinase pathways inhibitor, represents a valid etoposide-sparing drug that showed association with favourable outcomes in association with steroids.

Although a previous review (9) highlighted the importance of this syndrome in critical area, specific and updated evidence supporting personalized diagnostic and therapeutic strategies for ICU is still lacking (1). For these reasons, we consider the results of the present work of particular interest in the context of intensive care, since HLH is a condition classically treated and studied by hematologist who developed a strong diagnostic awareness and standardized treatment protocols. Furthermore, the differential diagnosis of this condition in the critical care context is typically septic shock and/or MOD that accounts for a vast majority of ICU population.

Considering the overall review, our analysis appears to highlight a decidedly significant incidence of HLH, which is progressively increasing over time, in step with the diagnostic awareness of the treating physicians. Diagnostic score, such as HScore, can rapidly lead to HLH diagnosis with a more than acceptable level of certainty even in critically ill patients making HLH rule out easy and feasible when assessing most critical ICU patients.

The growing relevance of laboratory tests—triglycerides, ferritin, etc.—in new diagnostic protocols represents another great clinical clue. In fact, although invasive diagnostic methods are certainly difficult, particularly in critical patients, and require more time for reporting, the combination of clinical and laboratory diagnosis scores represents a feasible option that should be highlighted. Soluble IL2 receptor (CD25) is a simple and under-utilized biomarker for T cell activation in HLH and its use should be encouraged in HLH diagnostic work up. CD25 is also useful for differentiating catastrophic Still’s disease from HLH, as both condition could possibly need intensive care support. However, in our review, we found that these laboratory tests were explicitly reported only in a minority of cases, possibly suggesting how they still need to be implemented in clinical practice.

**Figure 2:**
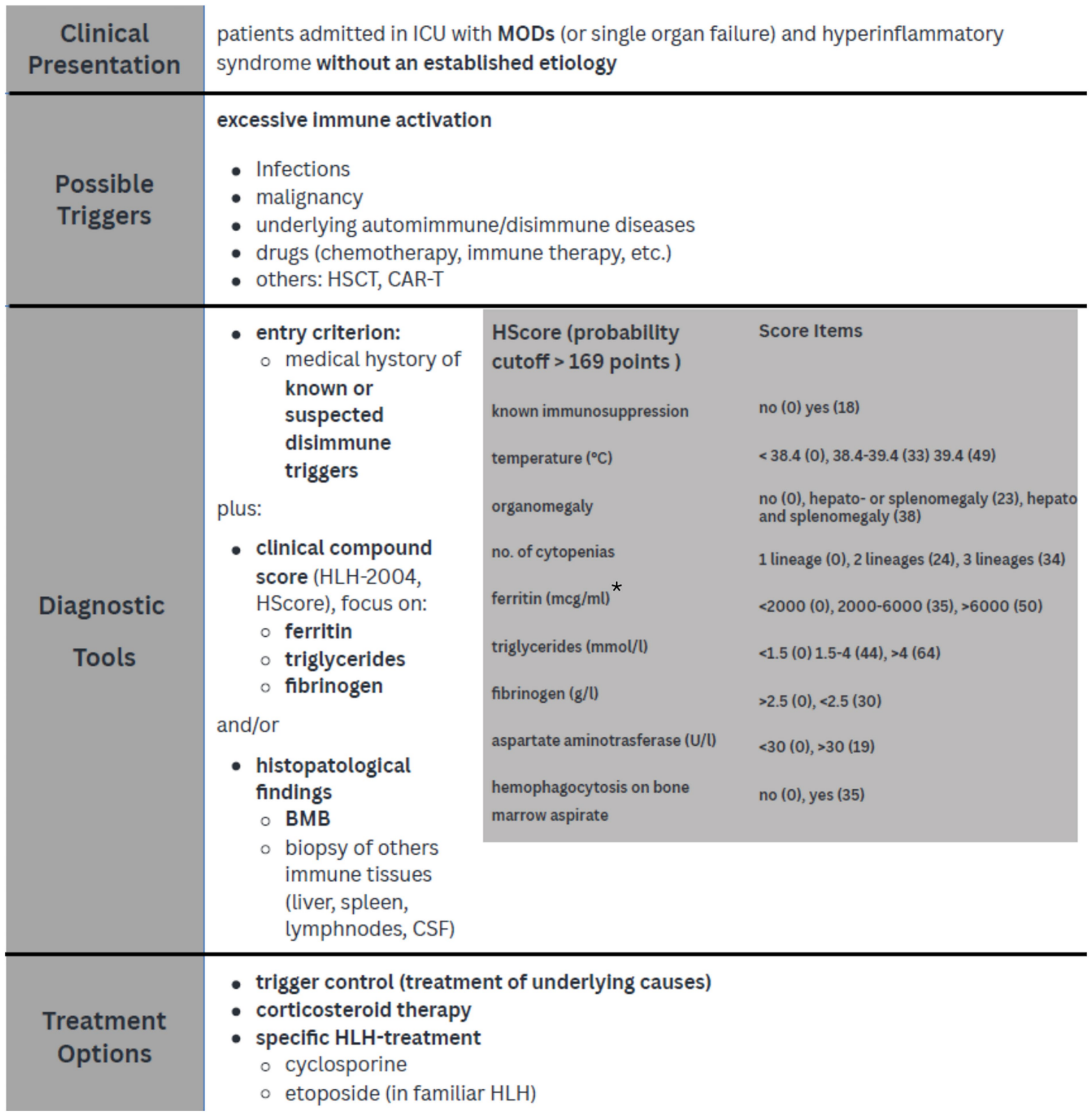
Diagnostic approach and treatment strategies in critically ill patients with HLH. *Knaak et al. demonstrated that a ferritin level > 3,000 μg/L improved diagnostic power when associated with HLH-2004 score; Lachmann et al. suggest that a threshold of 9,000 μg/L is very sensible and specific for HLH diagnosis in ICU SIRS sistemic inflammatory response syndrome; MODs multi organ dysfunction; HSCT hematopoietic stem cell transplantation, CAR-T chimeric antigen receptor T-cell, BMB bone marrow biopsy, CSF cerebrospinal fluid.

Data from this literature review show how diagnostic score, such as HScore, can rapidly lead to HLH diagnosis with a more than acceptable level of certainty even in critically ill patients (4, 10). A possible diagnostic and treatment approach for HLH, applicable in the ICU setting, is suggested in Figure 2.

## Limitations

Our study has several limitations. First, the narrative (non-systematic) approach to data selection, the absence of formal bias assessment, and the inherent limitations of using uncontrolled observational data. In fact, all papers included in our analysis are case report or case series, with risk of survival bias even if overall ICU mortality is in line with other studies about HLH in critical care population.

Given the nature of the included studies, we did not apply formal risk of bias tools, which are typically designed for comparative or interventional studies.

Furthermore, we decided to include in this analysis only articles published after 2014 when HScore has been proposed for HLH diagnosis, to underline the importance of the proposed score and the impact on clinical diagnosis. However, this decision might limit the number of the included articles.

Finally, due to lack of standardized medical treatment for HLH in critical care population, we found strong differences in treatment strategies among different reports, for example in steroid use with different molecules and different regimens, limiting the strength of the treatment indications.

## Conclusion

HLH is a syndrome characterized by immune dysregulation and hyperinflammation that can present with multi-organ disfunction. Prompt diagnosis in ICU is particularly challenging due to critical presentation, limited diagnostic options and clinical awareness.

Given that several HLH trigger are extremely frequent in critical patients, HLH should always be considered in patients with MOD or sepsis/septic shock-like clinical presentation without an established etiology and predisposing risk factors for HLH.

This narrative review is deliberately based on case reports and case series, with the aim of evaluating the available ICU-specific literature, limited, but of growing relevance. Despite the underlined limitations, some aspects of great clinical relevance emerge, in particular the association of SARS-CoV-2 and hematologic malignancies with mortality. Diagnostic scores such as HScore or HLH-2004 can help clinicians in identifying HLH especially with the combination of non-invasive laboratory tests, but the relevance of the role of bone marrow biopsy, remain pivotal. In the absence of effective treatment strategies, which continue to be mutuated from hematologic populations, further studies are needed to develop treatment protocols tailored on critical care population.

## Data Availability

The original contributions presented in the study are included in the article/supplementary material, further inquiries can be directed to the corresponding author.
